# Maternal Exposure to Non-nutritive Sweeteners Impacts Progeny’s Metabolism and Microbiome

**DOI:** 10.3389/fmicb.2019.01360

**Published:** 2019-06-20

**Authors:** Stephanie Olivier-Van Stichelen, Kristina I. Rother, John A. Hanover

**Affiliations:** ^1^National Institute of Diabetes and Digestive and Kidney Diseases, National Institutes of Health, Bethesda, MD, United States; ^2^Department of Biochemistry, Medical College of Wisconsin, Milwaukee, WI, United States

**Keywords:** microbiome, non-nutritive sweeteners, sucralose, acesulfame-K, metabolism, pregnancy, lactation

## Abstract

Non-nutritive sweeteners (NNS) are marketed as sugar alternatives providing sweet taste with few or no calories. Yet their consumption has been linked to metabolic dysfunction and changes in the gut microbiome. NNS exposure mostly originates from diet beverages and sweetener packages in adults or breastmilk in infants. Consequences of early life exposure remain largely unknown. We exposed pregnant and lactating mice to NNS (sucralose, acesulfame-K) at doses relevant for human consumption. While the pups’ exposure was low, metabolic changes were drastic, indicating extensive downregulation of hepatic detoxification mechanisms and changes in bacterial metabolites. Microbiome profiling confirmed a significant increase in firmicutes and a striking decrease of *Akkermansia muciniphila*. Similar microbiome alterations in humans have been linked to metabolic disease and obesity. While our findings need to be reproduced in humans, they suggest that NNS consumption during pregnancy and lactation may have adverse effects on infant metabolism.

## Introduction

Non-nutritive sweeteners such as sucralose and acesulfame-K are highly prevalent in the diet of United States adults and children (Sylvetsky and Rother, 2016). This is in part due to growing awareness of sugar’s adverse health effects. Yet, multiple clinical studies have reported similar detrimental health effects related to NNS as seen with sugar, even when NNS are used at doses below the acceptable daily intake (ADI) limit determined by the FDA (Swithers, 2013). Herein are some of the plausible mechanisms explaining this apparently paradoxical relationship. For example, ingestion of a NNS sweetened products elicits less reward response in the central nervous system than sugar sweetened foods or beverages (Tellez et al., 2016). NNS also alter incretin and insulin secretion (Jang et al., 2007; Mace et al., 2007; Margolskee et al., 2007; Brown and Rother, 2012), and may upregulate pro-inflammatory and adipogenesis promoting pathways (Simon et al., 2013; Bian et al., 2017a). NNS also impact glucose metabolism (Abou-Donia et al., 2008) and the gut microbiome (Abou-Donia et al., 2008; Cowan et al., 2013; Palmnäs et al., 2014; Suez et al., 2014; Bian et al., 2017b).

However, NNS are still widely used despite missing critical experimental data ensuring their safety. First, none of the existing literature investigated the effect of combined sweeteners exposure, when this is mostly the case in commercial products. Secondly, high-dose NNS diets are often administered in animal models, reducing the relevance of those studies for human health. Finally, least is known about the consequences of NNS use during pregnancy and lactation (American Dietetic Association, 2004).

Human exposure to NNS begins early through breastmilk, infant rehydration solutions and medications (Freedman et al., 2010; Sylvetsky et al., 2015). To date, a higher infant BMI at 1 year of age has been associated with maternal NNS consumption during pregnancy in a recent epidemiology study (Azad et al., 2016). Studies in animals suggest that acesulfame-K crosses the placenta, and *in utero* exposure results in increased sweet preference in adulthood (Zhang et al., 2011; Chen et al., 2013). In other animal studies, sucralose exposure during pregnancy showed no deleterious effects on fetal organogenesis (Kille et al., 2000) but increased the risk of hematopoietic neoplasia in male offspring (Soffritti et al., 2016).

In light of the reported NNS effects on the gut microbiome (Abou-Donia et al., 2008; Cowan et al., 2013; Palmnäs et al., 2014; Suez et al., 2014; Bian et al., 2017b) and metabolism (Brown and Rother, 2012; Cowan et al., 2013; Simon et al., 2013; Swithers, 2013; Palmnäs et al., 2014; Suez et al., 2014; Bian et al., 2017b), it is important to investigate the relationship between NNS consumption in pregnancy and lactation and the offspring’s microbiome and metabolism. Indeed, transmission of the microbiome from mother to child is a key factor, which influences metabolic regulation from early on, and is related to an increased risk of obesity, asthma and celiac disease (Faith et al., 2013; Bäckhed et al., 2015). In this study, we exposed pregnant and lactating mice to a mixed solution of sucralose and acesulfame-K at physiological concentrations and observed the impact on pre-weaned offspring (d20). Despite the low exposure through breastmilk, pups showed major alterations of metabolites in plasma and feces. In addition to amino acid alterations, deregulation of hepatic detoxification occurred in pups exposed to NNS. The resulting inefficient liver clearance and increased oxidative stress may have deleterious consequences as pups are challenged nutritionally during weaning and beyond. In addition to major bacterial metabolite variation, an increase of firmicutes as well as loss of one major beneficial species, *Akkermansia muciniphila*, were confirmed by microbiome sequencing. For the first time, we have shown that pre- and post-natal exposure (∼40 days) to NNS impacts the offspring’s metabolism and microbiome, predictive of relevant future metabolic dysregulation.

## Materials and Methods

### Mice

C57Bl6 mice (Jackson Laboratory) were ordered at 8 weeks of age and acclimated in the mouse facility for 2 weeks. Mice were maintained in a 12-h light/dark cycle with *ad libitum* water and regular NIH-07 chow diet (22.5% protein, 4.5% fat, 4.5% fiber). The animals were maintained and the experiment performed according to the animal protocols approved by the NIDDK Animal Care and Use Committee (NIH) (#K023-LCBB-16).

**FIGURE 1:**
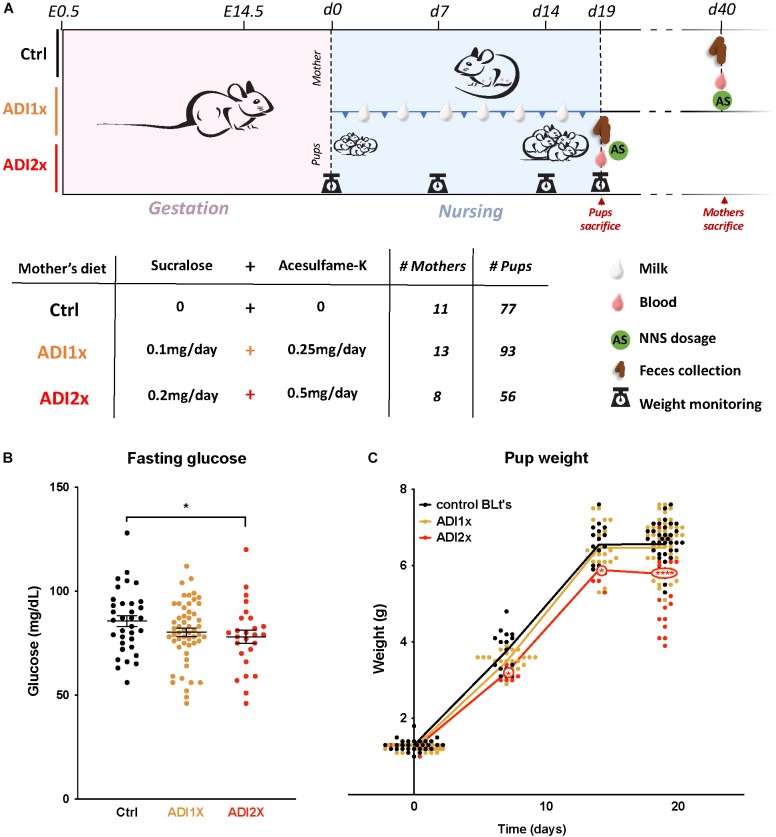
Pups born to NNS-exposed mothers have observable metabolic phenotypes. **(A)** Experimental design: Mothers were fed starting on day 0.5 post-coitus (E0.5) until sacrifice. Two different concentrations of sucralose and acesulfame-K (ADI1x and ADI2x) were used for the experiments. Pups were sacrificed on postnatal day 20 (d20), and mothers were sacrificed after. All mice were fasted, weighed and blood was drawn before sacrifice. **(B)** Fasting glucose of 20-day old pups was measured before sacrifice (*n* = 30) (ANOVA). **(C)** Pups’ weights were monitored from d1 to d20 and significance was analyzed using ANOVA (*n* = 30). Significance values: ^∗∗∗∗^*p* < 0.001, ^∗^*p* < 0.05.

Mice were single-housed 1 week before the experiment. Pregnancy of C57Bl6 mice was assessed by visualization of the vaginal plug and defined as E0.5. Pregnant mice were fed a quarter pellet/day of Rodent BLT’s^TM^ (Bio-serv) onto which were pipetted either: (1) water (Ctrl); (2) 10 μL of water with 0.1 mg of sucralose and 0.25 mg of acesulfame-K [equivalent to upper limit of ADI for human consumption (ADI1x)]; (3) 20 μL of water with 0.2 mg of sucralose and 0.5 mg of acesulfame-K [twice the ADI (ADI2x)] (Figure 1A). The ADI for sucralose and acesulfame-K are 5 and 15 mg/kg of bodyweight/day, respectively. Pellets were added into a plastic cup in the cage every day and supplemented the regular chow diet. Pellet ingestion was assessed visually every day.

One day prior to sacrifice, mice were fasted overnight (16 h). Glycaemia was measured at the mouse tail using a Contour apparatus (Bayer). Mice were sacrificed after isoflurane anesthesia (Baxter).

### Mouse Sample Collection

Retro-orbital blood collection was performed under isoflurane anesthesia.

Following sacrifice, the entire digestive tract from stomach to rectum was dissected and flushed with 10 mL of phosphate buffer saline (PBS) buffer. The gut flush was then concentrated by collecting the retained fraction on 0.22 μm filter paper.

Milk collection was performed on gestational day 15. The litter was separated from the mother 2 h prior to milking. Under isoflurane anesthesia, mice were injected intraperitoneally with 2IU of oxytocin (VetOne). After a few minutes, milk was collected by squeezing each mammary gland (Willingham et al., 2014). All samples were flash-frozen and stored at -80°C.

### NNS Concentrations

Sucralose and acesulfame-K concentrations in plasma, urine, feces and breast milk were measured in triplicate using liquid chromatography-mass spectrometry (LC-MS). Analyses were performed with an Acquity I-Class UPLC (Waters Corp., Milford, MA, United States) and an Acquity UPLC BEH C-18 column (2.1 mm × 50 mm, 1.7 μm) coupled with a Q-Exactive MS (Thermo Scientific, Waltham, MA, United States) with a HESI-II electrospray source. Analyses were performed using isotopically labeled sucralose as the internal standard for sucralose measurements and isotopically labeled acesulfame-K for acesulfame-K measurements. The %CV for the samples throughout experiments was 4.5% for sucralose and 0.8% for acesulfame-K, respectively, and for samples between days was 5.8% for sucralose and 1.0% for acesulfame-K, respectively.

#### Immunohistochemistry

Liver were sectioned, paraffin-embedded and stained by Histoserv. Inc., (Germantown, MD). Control stain were performed using heart (Perl), liver with gall bladder (Hall) or liver (PAS). Digestion of glycogen was performed in combination with PAS staining.

### Metabolomics

A non-targeted metabolomics analysis was performed on feces and plasma samples by Metabolon^®^.

#### Sample Preparation

Samples were prepared using the automated MicroLab STAR^®^ system from Hamilton Company. Several recovery standards were added prior to the first step in the extraction process for quality control purposes. To remove protein, to dissociate small molecules bound to protein or trapped in the precipitated protein matrix, and to recover chemically diverse metabolites, proteins were precipitated with methanol under vigorous shaking for 2 min (Glen Mills GenoGrinder 2000) followed by centrifugation. The resulting extract was divided into five fractions: two for analysis by two separate reverse phase (RP)/UPLC-MS/MS methods with positive ion mode electrospray ionization (ESI), one for analysis by RP/UPLC-MS/MS with negative ion mode ESI, one for analysis by HILIC/ ultra-performance liquid chromatography (UPLC)-MS/MS with negative ion mode ESI, and one sample was reserved for backup. Samples were placed briefly on a TurboVap^®^ (Zymark) to remove the organic solvent. The sample extracts were stored overnight under nitrogen before preparation for analysis.

#### QA/QC

Several types of controls were analyzed in concert with the experimental samples: a pooled matrix sample generated by taking a small volume of each experimental sample (or alternatively, use of a pool of well-characterized human plasma) served as a technical replicate throughout the data set; extracted water samples served as process blanks; and a cocktail of QC standards that were carefully chosen not to interfere with the measurement of endogenous compounds were spiked into every analyzed sample, allowed instrument performance monitoring and aided chromatographic alignment. Instrument variability was determined by calculating the median relative standard deviation (RSD) for the standards that were added to each sample prior to injection into the mass spectrometers. Overall process variability was determined by calculating the median RSD for all endogenous metabolites (i.e., non-instrument standards) present in 100% of the pooled matrix samples. Experimental samples were randomized across the platform run with QC samples spaced evenly among the injections.

#### Ultrahigh Performance Liquid Chromatography-Tandem Mass Spectroscopy (UPLC-MS/MS)

All methods utilized a Waters ACQUITY UPLC and a Thermo Scientific Q-Exactive high resolution/accurate mass spectrometer interfaced with a heated electrospray ionization (HESI-II) source and Orbitrap mass analyzer operated at 35,000 mass resolution. The sample extract was dried then reconstituted in solvents compatible to each of the four methods. Each reconstitution solvent contained a series of standards at fixed concentrations to ensure injection and chromatographic consistency. One aliquot was analyzed using acidic positive ion conditions, chromatographically optimized for more hydrophilic compounds. In this method, the extract was gradient eluted from a C18 column (Waters UPLC BEH C18-2.1 × 100 mm, 1.7 μm) using water and methanol, containing 0.05% perfluoropentanoic acid (PFPA) and 0.1% formic acid (FA). Another aliquot was also analyzed using acidic positive ion conditions, however, it was chromatographically optimized for more hydrophobic compounds. In this method, the extract was gradient eluted from the same afore mentioned C18 column using methanol, acetonitrile, water, 0.05% PFPA and 0.01% FA and was operated at an overall higher organic content. Another aliquot was analyzed using basic negative ion optimized conditions using a separate dedicated C18 column. The basic extracts were gradient eluted from the column using methanol and water, however, with 6.5 mM ammonium bicarbonate at pH 8. The fourth aliquot was analyzed via negative ionization following elution from a HILIC column (Waters UPLC BEH Amide 2.1 × 150 mm, 1.7 μm) using a gradient consisting of water and acetonitrile with 10 mM ammonium formate, pH 10.8. The MS analysis alternated between MS and data-dependent MS^n^ scans using dynamic exclusion. The scan range varied slighted between methods but covered 70–1000 m/z. Raw data files are archived and extracted as described below.

#### Bioinformatics

The informatics system consisted of four major components, the Laboratory Information Management System (LIMS), the data extraction and peak-identification software, data processing tools for QC and compound identification, and a collection of information interpretation and visualization tools for use by data analysts. The hardware and software foundations for these informatics components were the LAN backbone, and a database server running Oracle 10.2.0.1 Enterprise Edition.

#### Data Extraction and Compound Identification

Raw data was extracted, peak-identified and QC processed using Metabolon’s hardware and software. These systems are built on a web-service platform utilizing Microsoft’s .NET technologies, which run on high-performance application servers and fiber-channel storage arrays in clusters to provide active failover and load-balancing. Compounds were identified by comparison to library entries of purified standards or recurrent unknown entities. Metabolon maintains a library based on authenticated standards that contains the retention time index (RI), mass to charge ratio (*m/z)*, and chromatographic data (including MS/MS spectral data) on all molecules present in the library. Furthermore, biochemical identifications are based on three criteria: retention index within a narrow Resonance Ionization (RI) window of the proposed identification, accurate mass match to the library +/-10 ppm, and the MS/MS forward and reverse scores between the experimental data and authentic standards. The MS/MS scores are based on a comparison of the ions present in the experimental spectrum to the ions present in the library spectrum. While there may be similarities between these molecules based on one of these factors, the use of all three data points can be utilized to distinguish and differentiate biochemicals. More than 3300 commercially available purified standard compounds have been acquired and registered into LIMS for analysis on all platforms for determination of their analytical characteristics. Additional mass spectral entries have been created for structurally unnamed biochemicals, which have been identified by their recurrent nature (both chromatographic and mass spectral). These compounds have the potential to be identified by future acquisition of a matching purified standard or by classical structural analysis.

#### Curation

A variety of curation procedures were carried out to ensure that a high-quality data set was made available for statistical analysis and data interpretation. The QC and curation processes were designed to ensure accurate and consistent identification of true chemical entities, and to remove those representing system artifacts, mis-assignments, and background noise. Metabolon data analysts use proprietary visualization and interpretation software to confirm the consistency of peak identification among the various samples. Library matches for each compound were checked for each sample and corrected if necessary.

#### Metabolite Quantification and Data Normalization

Peaks were quantified using area-under-the-curve. For studies spanning multiple days, a data normalization step was performed to correct variation resulting from instrument inter-day tuning differences. Essentially, each compound was corrected in run-day blocks by registering the medians to equal one (1.00) and normalizing each data point proportionately (termed the “block correction”). For studies that did not require more than one day of analysis, no normalization is necessary, other than for purposes of data visualization. In certain instances, biochemical data may have been normalized to an additional factor (e.g., cell counts, total protein as determined by Bradford assay, osmolality, etc.) to account for differences in metabolite levels due to differences in the amount of material present in each sample.

### Liver Transcriptomics

RNeasy Mini Kit (Qiagen) was used to extract RNA from 100 mg of liver. Four microliter of qScript cDNA Supermix (Quanta bioscience) was added to 500 μg of RNA extract and run on a thermocycler to provide cDNA using the following program: 25°C/5 min; 42°C/30 min; 85°C/5 min. cDNA quantity and quality were then assessed using nanodrop spectrophotometer. cDNA samples were analyzed with RT^2^ Profiler PCR Array- Mouse Drug Metabolism: Phase I Enzymes or Mouse Drug Metabolism: Phase II Enzymes (Qiagen) according to the manufacturer protocol. Samples were processed on 7900HT Fast Real-Time PCR system (Applied Biosystems).

### Microbiome

Feces DNA samples were obtained from gut flushes (see sample collection) using the Fast DNA Spin Kit feces (MPBio). DNA quantity and quality were then assessed using a Nanodrop spectrophotometer.

Library preparation for sequencing of the V3-V4 region of 16S ribosomal DNA was performed using two PCRs. First, 16S DNA was amplified using 1 μg of feces DNA, 2× MyTaq Red Mix (Bioline) and the following primers: 16S Forward: TCGTCGGCAGCGTCAGATGTGTATAAGAGACAGCCTACGGG NGGCGGCWGCAG; 16S Reverse: GTCTCGTGGGCTCGGAGATGTGTATAAGAGACAGGACTACH VGGGTATCTAATCC. Amplification was performed on a thermocycler using the following program: 95°C/3 min; 95°C/30 s; 55°C/30 s; 72°C/30 s (repeat three last steps 35×); 72°C/5 min; 4°C/∞. To assess PCR quality, samples were run on 2% agarose E-gel (Invitrogen). Then, 2 μL of PCR product was used as a matrix for the second “adapters” PCR. Each 16S amplicon was then amplified using a unique couple of barcode-containing primers for dual-indexing of samples during the sequencing process (Fadrosh et al., 2014). Addition of the adapters on 16S amplicons was checked on 2% agarose gel. Finally, amplicon bands were extracted from agarose gels using QIAquick Gel Extraction Kit (Qiagen). This step cleaned up the final PCR product and removed eventual high molecular weight primer dimers, thus increasing the sequencing yield. A mixed sample of about 30 samples (1:1 ratio) was used for sequencing.

Sequencing was performed by the sequencing core (NIDDK- National Institutes of Health) using MiSeq Reagent Kit v2 (600-cycle) (Illumina).

Sequencing output was first de-multiplexed and sequences were then analyzed with macQIIME workflow in “open reference OTU picking” protocol. Alpha- and Beta-diversity (Principal Coordinate Analysis- PCoA) were performed on R Studio using the phyloseq package.

### *Akkermansia muciniphila* Quantitative PCR

Two sets of primers were used to determine the quantity of *A. muciniphila* in feces samples (5→3): *A. muciniphila* Forward: CAGCACGTGAAGGTGGGGAC; *A. muciniphila* Reverse: CCTTGCGGTTGGCTTCAGAT; 16S Forward: TCGTCGGCAGCGTCAGATGTGTATAAGAG ACAGCCTACGGGNGGCGGCWGCAG; 16S Reverse: GTCTCGTGGGCTCGGAGATGTGTATAAG AGACAGGACTACHVGGGTATCTAATCC. One μg of DNA was mixed with fast SYBR Green mastermix (Applied Biosystems), two primers (100 μM) and amplified on 7900HT Fast Real-Time PCR system (Applied Biosystems). Each determination was performed in triplicate and *A. muciniphila* abundance was measured relative to 16S expression.

### Biostatistics

Spearman correlation, *t*-test and ANOVA analysis, Principal Component Analysis and Volcano plots were performed and plotted using R. The scripts for the analysis were posted in GITHUB^1^ .

### Akkermansia muciniphila

*Akkermansia muciniphila* was purchased from ATCC (BAA-835) and maintained under anaerobic condition at 37°C either in Remel PRAS Brain Heart Infusion Broth (Thermo Scientific) or on Trypticase Soy Agar plates with Sheep Blood (BD BBL). *A. muciniphila* growth experiment was conducted in 96-well plate liquid culture by measuring the absorbance at 600 nm on the POLARstar Omega spectrophotometer (BMG Labtech). Media was supplemented with 100 μg/mL of ampicillin, 100 ng/mL of acesulfame-K and 35 ng/mL of sucralose (“low NNS”) or 100 μg/mL of acesulfame-K and 35 μg/mL of sucralose (“high NNS”).

### Public Repository

Microbiome and metabolomics data have been published on Open Science Framework (OSF)^2^.

All scripts for analysis and/or plotting in this article have been published on GitHub^1^.

## Results

### Mothers Transmitted NNS Through Breast Milk Leading to Lower Fasting Glucose and Weight in Pups

In brief, C57Bl6 mice (*n* = 31) were fed either water (control), or a combination of sucralose and acesulfame-K at the ADI (Sucralose ADI: 5 mg/kg/day; Acesulfame-K ADI: 15 mg/kg/day) or twice the ADI (ADI2x) during pregnancy and lactation (Figure 1A). NNS concentrations were measured in mothers’ samples (blood, feces, breastmilk) after fasting and sacrifice post-weaning (Supplementary Figures S1A,B and Supplementary Table S1). While no NNS were observed in the control group, NNS were found in mothers’ blood and feces in both NNS groups. As expected, based on previous pharmacokinetic studies, sucralose was mainly found in mother’s feces whereas acesulfame-K was found in equal amounts in feces and blood. Both sweeteners were found in breastmilk (d15) at considerably lower concentrations than in blood and/or feces, and concentrations were highly variable between individual animals. A total of 226 20-day old pups distributed across the 3 groups were analyzed in the present study. Pups’ blood and feces samples were collected at d20 after fasting and sacrifice (Supplementary Figures S1C,D and Supplementary Table S1). Overall, both NNS were either unmeasurable or present at low concentrations in the pups’ blood and feces compared to their mother’s. Sucralose was found mostly in the feces and was roughly correlated with the amount of sucralose in mothers’ breast milk. Acesulfame-K concentrations were below the detection limit in pups’ blood and feces, but measurable in the pups’ urine when analyzed in newborns and 14 day-old pups (Supplementary Figure S1E and Supplementary Table S1). These measurements confirmed that both NNS were transmitted prenatally. Litter size and sex distribution were comparable in NNS *vs.* Ctrl pups (Supplementary Table S2). Measurement of the pups’ fasting glucose at d20 showed a decrease in ADI2x pups (*p* = 0.02) compared to water, while a non-significant difference was observed between the ADI1x and Ctrl groups (*p* = 0.16) (Figure 1B). The weight of the ADI2x pups was also reduced compared to Ctrl animals with weight curves gradually separating in the first week of life (Figure 1C).

### Metabolomics Analysis Revealed Alterations in Amino Acid Metabolism, Detoxification Pathways and Microbiome-Linked Metabolites in NNS Pups

**FIGURE 2:**
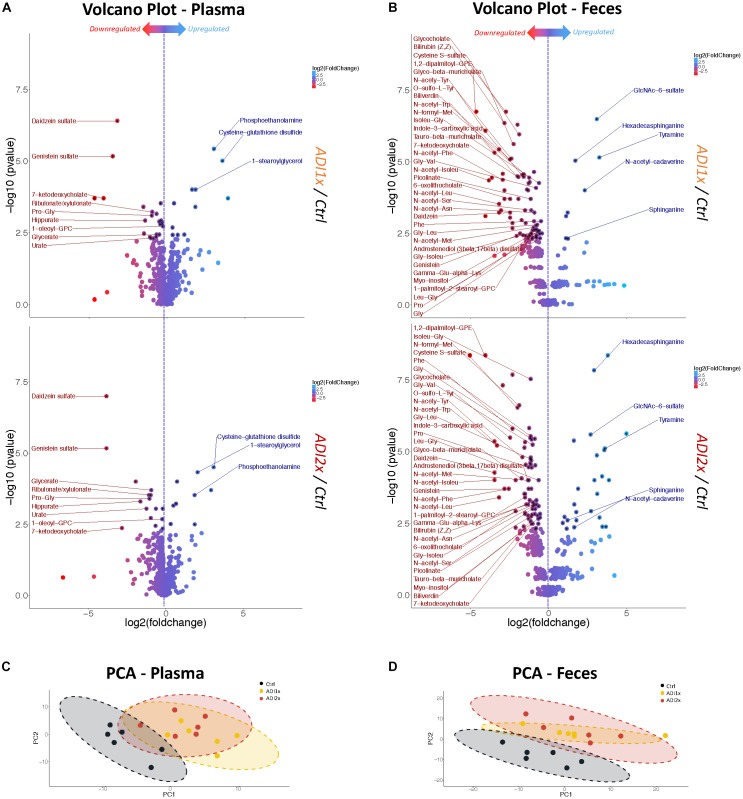
Feces and plasma samples highlight major metabolic deregulation in pups born to NNS-exposed mothers. **(A,B)** Volcano plot of metabolites in plasma and feces for ADI1x (top panel) and ADI2x (bottom panel) compared to control group (*n* = 6). Significant metabolites (*p* <= 0.005) are pointed to with a black asterisk. Only highly significant deregulated metabolites (*p* <= 0.005) in both ADI1x and ADI2x in plasma or feces are labeled on volcano plots (ANOVA). **(C,D)** Principal component analysis (PCA) for plasma and fecal metabolites. Ellipses represent Student’s *t*-distribution (*n* = 6).

To further define the consequences of the NNS exposure, we analyzed both feces and plasma metabolites in the pups using a non-targeted metabolomics approach. The analysis highlighted 594 and 708 metabolites in plasma and feces, respectively, across 18 samples, (6 in each group). In the NNS pups’ plasma, more than 100 metabolites were deregulated compared to the Ctrl pups and almost 200 were deregulated in feces at *p* <= 0.05 threshold (Supplementary Figure S2). Volcano plots representing changes of both ADI1x and ADI2x *vs.* control group revealed extensive metabolic deregulation in both NNS groups (Figure 2A,B and Supplementary Tables S3, S4). Compared to Ctrl, NNS feces samples showed the most deregulated metabolites (at least 5 up and 35 down) compared to plasma (at least 3 up and 9 down) for both ADI1x and ADI2x pups. All three groups of pups separated significantly in a principal component analysis (PCA) and by ADONIS test (feces *r*^2^ = 0.30 and *p* < 0.001; plasma *r*^2^ = 0.28 and *p* < 0.001), highlighting distinct metabolomes (Figure 2C,D). Of note, a pool of metabolites was uniquely deregulated in ADI2x compared to ADI1X (plasma: 59 ups and 28 downs; feces: 107 up and 58 down), suggesting dose-dependent metabolic deregulation.

**FIGURE 3:**
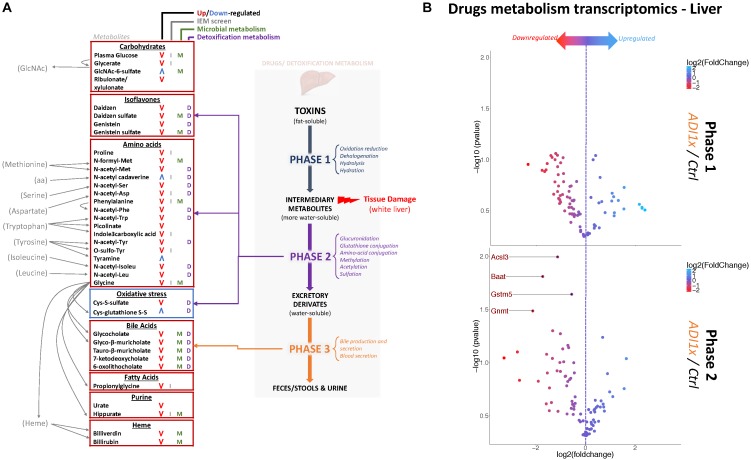
Non-nutritive sweetener pups exhibit an imbalanced liver detoxification system. **(A)** A summary of highly deregulated metabolites and their interplay with detoxification/drug metabolism. IEM, Inborn Error of Metabolism **(B)** Volcano plots representing the fold change in mRNA levels of phase 1 (Top panel) and phase 2 (Bottom panel) metabolic enzymes from livers of 20-day old control and ADI1x pups are shown. Labeled genes are significantly deregulated in ADI1x *vs.* control pups (*p* <= 0.05) (*t*-test).

Amino acid metabolism was the most deregulated class of metabolites in NNS pups (Figure 3A, 2A,B and Supplementary Tables S3, S4). Along with primary amino acid metabolites, numerous acetylated, sulfated or methylated amino acids as well as products of amino acid metabolism (picolinate, indole-3-carboxylic acid) were downregulated in NNS pups; N-acetyl cadaverine and tyramine, products of amino acid breakdown, were highly upregulated. Many glycine-containing dipeptides were also significantly decreased in NNS pups. Similarly, numerous products of glycine metabolism were downregulated such as bile acids, heme breakdown products, hippurate and propionylglycine.

Both carbohydrate and lipid metabolism were found to be altered in the NNS pups (Figure 3A, 2A,B and Supplementary Tables S3, S4). The presence of sucralose as well as the decreased plasma glucose levels in NNS fed pups were also confirmed by metabolomics. GlcNAc-6-sulfate was at least 6 times higher in pups’ feces in both NNS groups, suggesting a glycosaminoglycan processing deficiency following NNS exposure. In the lipids, modified glycerylphosphorylcholine and glycerylphosphoethanolamine as well as myoinositol were significantly downregulated in NNS exposed pups while structural lipids such as phosphoethanolamine, hexadecasphinganine and sphinganine were upregulated.

Numerous deregulated metabolites were associated with improper liver clearance and detoxification pathways (Figure 3A, 2A,B and Supplementary Tables S3, S4). Of note, both primary and secondary bile acids, N-acetylated amino acids and sulfated metabolites were downregulated in NNS pups. In addition, accumulation of cys-glutathione disulfide was consistent with unprocessed reactive intermediary metabolites normally removed through phase 2 detoxification (Figure 3A).

### NNS Exposure Was Associated With Deregulation of Hepatic Phase 2 Detoxification, Bile Acids and a Pale Liver Phenotype

Metabolic detoxification occurs primarily in the liver and is divided into 3 phases: transformation, conjugation and transport (Almazroo et al., 2017) (Figure 3A). To confirm the unbiased metabolomics findings that NNS pups have altered detoxification metabolism, gene expression was analyzed in the livers of control and ADI1x pups by phase 1 and 2 drug metabolism qPCR array (Figure 3B and Supplementary Tables S5, S6). Transcriptomics of 20-day old pups’ livers showed downregulated transcripts encoding enzymes in phase 2 metabolism. Indeed, the bile acid-CoA: amino acid N-acetyl transferase Baat, the acyl-CoA synthetase Acsl3, the glycine modifying enzyme Gnmt and glutathione S-transferase Gstm5 were highly downregulated. These transcriptional results are consistent with improper phase 2 detoxification and accumulation of intermediary metabolites. Furthermore, whitening of NNS pups’ livers was consistently observed in the ADI2x group and to a lesser degree in the ADI1x group (Supplementary Figure S3A). Intriguingly, common histologic analysis of the NNS livers did not reveal macroscopic damage to the liver (Supplementary Figure S3B – H&E and Masson). By immunohistochemistry analysis, there was no evidence of either altered glycogen (PAS), lipid droplets (H&E), iron (Perl) or bile (Hall) storages in the offspring of NNS exposed mothers (Supplementary Figure S3B).

### NNS Pups Exhibited Major Changes in Gut Microbiota

In addition to major changes in metabolism, metabolomics analysis identified deregulation of specific microbial metabolites (Figure 2, 3A). These observations suggested major changes in the microbiome in addition to amino acid metabolic deregulation in the NNS pups. Many bacterial metabolites (Gottschalk, 2012) differed in their abundance in NNS pups such as N-formyl-methionine, 6-oxolithocholate, 7-ketodeoxycholate, hippurate, daidzein and genistein. Considering the existing literature on NNS and adult microbiome, we hypothesized that NNS could also alter the pups’ gut microbiome.

**FIGURE 4:**
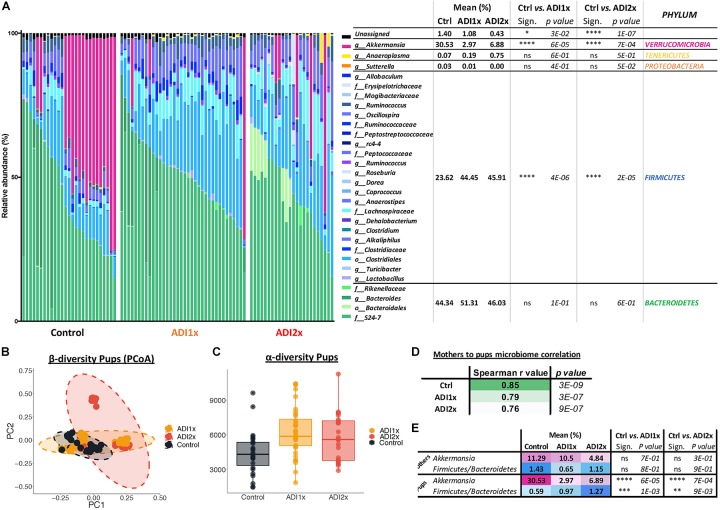
Major microbiome changes are observed in NNS pups. **(A)** Summary of 16S sequencing of pup’s control, ADI1x and ADI2x gut microbiota. The mean, significance and *p*-value are represented at the phylum level in the right panel (Kruskal–Wallis test) (*n* = 24). g_: genus; f_: family; o_: order. **(B)** A beta-diversity analysis (Principal Coordinate Analysis (PCoA- unweighted unifrac) of control, ADI1x and ADI2x microbiome sequencing (*n* = 24). **(C)** An alpha-diversity analysis [observed operational taxonomic units (OTUs)] of control, ADI1x and ADI2x microbiome sequencing (*n* = 24). **(D)** The Spearman correlation and *p*-value measures of mothers and pups’ microbiome (*n* = 24). **(E)** The changes observed in mothers and pups in verrucomicrobia levels and firmicutes/bacteroidetes ratio (Kruskal–Wallis test) (*n* = 24). Significance values: ^∗∗∗∗^*p* < 0.001, ^∗∗∗^*p* < 0.005, ^∗∗^*p* < 0.01, ^∗^*p* < 0.05.

Bacteroidetes, verrucomicrobia and firmicutes represented 44.34, 30.53, and 23.62% of the Ctrl pups’ microbiota, respectively (Figure 4A and Supplementary Table S7). In NNS pups, firmicutes doubled (ADI1x = 44.45%; ADI2x = 45.91%), including clostridiales families *lachnospiraceae* and *ruminococcaceae* (*g_Oscillospira*). In contrast, verrucomicrobia, represented by a single species *A. muciniphila*, was significantly depleted in NNS pups (ADI1x = 2.97%; ADI2x = 6.88%). These observations were confirmed by analysis of the beta-diversity principal coordinate analysis (PCoA), showing separation of Ctrl, ADI1x and ADI2x pups (Figure 4B). All three groups of pups also separated significantly by ADONIS test (Unweighted Unifrac: *r*^2^ = 0.07 and *p* < 0.001; Weighted Unifrac: *r*^2^ = 0.18, *p* < 0.001) (Supplementary Figure S4D). A significant increase in alpha-diversity was also observed for both NNS groups, suggesting an increased variety of the gut microbiota (Figure 4C and Supplementary Figure S4E). This may be attributed to the loss of the highly abundant *A. muciniphila* in the gut in combination with increase of the firmicutes species.

Offspring’s microbiome dysbiosis is often explained by changes in the mother’s microbiome. Indeed, the microbiota is shaped from birth by successive exposure to the mother’s vaginal, skin and breast milk microbiomes (Palmer et al., 2007). Consequently, the same phyla were found in the mothers’ and pups’ microbiome (Supplementary Figure S4A and Supplementary Table S8). Ctrl and NNS-fed mothers’ microbiota were not statistically different. Similarly, Ctrl and NNS mothers’ microbiomes did not separate on the alpha or beta-diversity analysis (PCoA plot) or by ADONIS test (Supplementary Figures S4B–E). Therefore, the divergence between Ctrl and NNS pups’ microbiota cannot be explained by an alteration in the mothers’ microbiota. Mother-to-pup’s transmission can be analyzed by calculation of Spearman correlation coefficients for the different groups. A decrease in correlation was observed with NNS exposure, suggesting that the divergence between mothers’ and pups’ microbiomes were due to increasing NNS concentrations (Figure 4D).

Alteration of NNS pups’ microbiome also suggested metabolic imbalance, which correlated with the metabolomic data. Indeed, an increased firmicutes/bacteroidetes ratio is associated with obesity (Ley et al., 2005) while depletion of *A. muciniphila* is frequently a sign of metabolic dysregulation (Everard et al., 2013; Schneeberger et al., 2015; Figure 4E).

### *Akkermansia muciniphila* Colonization Was Defective in NNS Pups

**FIGURE 5:**
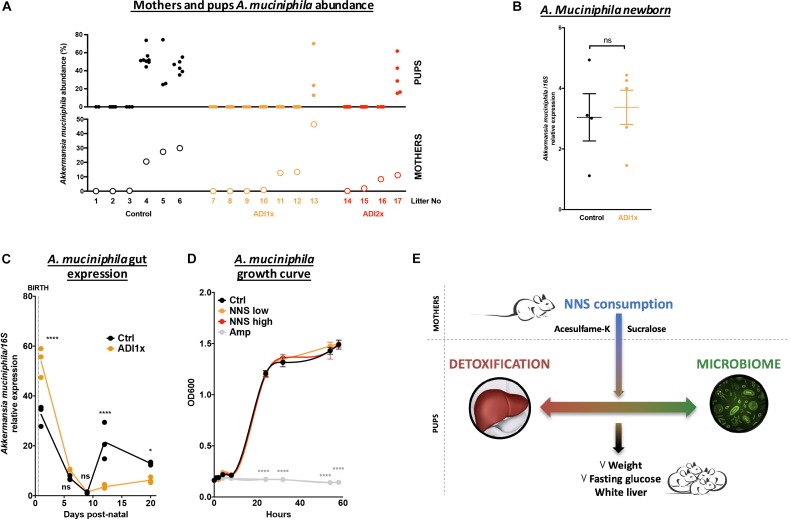
*Akkermansia muciniphila* is transmitted but does not consistently colonize pups born to NNS-exposed mothers. **(A)** *A. muciniphila* abundance analyzed by 16S sequencing (*n* = 4 litters) of pups (top panel) and mothers (bottom panel). **(B)** Control and ADI1x newborn gut DNA extraction analyzed by qPCR for *A. muciniphila*/16S (*n* = 4 newborns) (*t*-test). **(C)** Gut *A. muciniphila* expression analyzed by qPCR in control and ADI1x pups from d1 to d20 (ANOVA) (*n* = 5 pups, technical triplicate). **(D)** *A. muciniphila* growth curves measured by absorbance at 600 nm. PBS (control), sucralose and acesulfame-K (NNS) or ampicillin (Amp) were introduced into the culture media at 0 of the experiment (ANOVA) (*n* = 3). **(E)** Summary figure of the effect of sucralose and acesulfame-K consumption during pregnancy and lactation. Significance values: ^∗∗∗∗^*p* < 0.001, ^∗^*p* < 0.05.

A closer look at pups’ *A. muciniphila* level and their corresponding mothers by qPCR confirmed an improper transmission in NNS pups (Figure 5A). It is important to understand why *A. muciniphila* colonization was defective in NNS fed pups since this bacterium is widely regarded as beneficial (Everard et al., 2013; Schneeberger et al., 2015). However, *A. muciniphila* level analyzed by qPCR showed various levels single housed mothers 1 day before mating (Supplementary Figure S5). Breastfeeding is one of the major sources of bacteria and probiotics for the pups. Nevertheless, analysis of the newborn pups showed similar level of *A. muciniphila* in Ctrl and ADI1x animals right after first nursing (d1), suggesting that mothers properly transmitted *A. muciniphila* at birth to their pups (Figure 5B). However, pups’ *A. muciniphila* level over time (Figure 5C) showed an improper progression of colonization in the ADI1x group. Indeed, after the establishment of gut anaerobic environment (Pantoja-Feliciano et al., 2013), *A. muciniphila* colonized the Ctrl but not the ADI1x pups.

We then directly exposed *A. muciniphila* to various NNS concentrations or ampicillin and measured bacterial growth *in vitro* (Figure 5D). While ampicillin completely inhibited *A. muciniphila* growth, no difference was observed between control or NNS supplemented media suggesting that *A. muciniphila* can grow in the presence of NNS.

## Discussion

Use of NNS has greatly increased worldwide in the last few decades. While awareness of NNS related negative health outcomes is growing, critical knowledge gaps exist about the mechanisms and extent of NNS induced effects. Recently, a science advisory was released by the American Heart Association which encourages consumers to not replace sugar sweetened with artificially sweetened beverages but to focus on water (Johnson et al., 2009). The report also includes a description of research needs with specific mention of pediatric studies. Of note, government agencies usually prohibit the addition of NNS to baby and infant foods, but no specific recommendations exist for pregnant or lactating mothers. To fully understand NNS impact, it is necessary to conduct more studies early in life using a wider range of doses and NNS combinations. In this study, we investigated the effects of maternal consumption of a combined sweetener solution (sucralose and acesulfame-K) at concentrations relevant to human exposure, e.g., once or twice the ADI.

Gestating and lactating mouse mothers transmit the ingested NNS to the pups in utero, and through breastfeeding postnatally (McNeil Specialty Products Co., 1987; Zhang et al., 2011). Little is known about human in utero exposure, but transmission via breast milk has been established (Sylvetsky et al., 2015). The NNS exposed adult female mice showed no abnormalities. This is consistent with other studies in which NNS effects were typically observed after months of exposure (FDA, 1988, 1999). However, NNS pups were phenotypically different. In this study, we report significantly decreased weight and fasting glucose for the ADI2x pups. In experiments submitted to the FDA prior to sucralose’s approval, animals exposed to much higher concentrations had been found to have diminished weight which is mostly attributed to decreased food palatability (FDA, 1988, 1999). Another important observation was the hepatic discoloration, most evident in the ADI2x pups. Liver whitening can be observed in numerous conditions such as fatty liver disease, anemia [previously shown in sucralose exposure at pharmacologic doses (European Commission, 2000)], mitochondrial dysfunction and improper clearance of metabolites. Whereas these pathologies can take months to develop in rodents, NNS pups showed signs of whitening at only 20 days of life. Histologic analyses ruled out fatty liver disease and fibrosis and showed no specific pathology. Since macronutrient storage was not altered, we speculate that the cause of liver whitening is due to a combination of faulty detoxification, decreases in bile acid formation, increased oxidative stress, and decreased mitochondrial function.

### Metabolic Deregulation in NNS Pups

We found that NNS exposure of pregnant and nursing mothers causes critical changes in amino acid and detoxification metabolism in the progeny, identified by a non-targeted metabolomics analysis of feces and blood samples. Eleven of the significantly deregulated metabolites in NNS pups are part of standard screening tests for inborn errors of metabolism (IEM) in humans (El-Hattab, 2015). IEMs are mostly due to mutations in specific enzymes or transport proteins of important metabolic pathways. Frequently observed symptoms in IEM are growth failure, weight loss, developmental delay, liver failure and/or hypoglycemia, many of which are observed in the NNS exposed pups. Despite different etiologies (genetic mutation *vs.* environmental exposure), this suggests similarly interrupted processes of nutrient processing and detoxification.

Among the various deregulated metabolic pathways, the pups’ amino acid metabolism is most affected particularly the glycine metabolism. Glycine is used for synthesis of glutathione, bile acids, heme, hippurate and propionylglycine (Wang et al., 2013), all under-represented in the metabolomics data. Glycine shortage has been associated with suboptimal growth, impaired immune system function (Lewis et al., 2005) as well as many other conditions (Wijekoon et al., 2004). NNS pups also have a striking deficit in numerous compounds related to detoxification. A relationship with NNS has been suggested previously, demonstrated in adult rats by the upregulation of the multidrug resistance transporter P-gp and the cytochrome P450 Cyp3a4 and Cyp2d1 following sucralose exposure (Abou-Donia et al., 2008). In our hands, Cyp3a11, the mouse homolog of Cyp3a4, is also upregulated but did not reach statistical significance, potentially due to the short exposure. Furthermore, several phase 2 detoxification metabolism enzymes are downregulated in NNS pups. Among the deregulated enzymes is bile acid-CoA: amino acid N-acetyl transferase (Baat), which catalyzes the addition of glycine or taurine to unconjugated bile acids, consistent with the observed decrease of primary bile acids (O’Byrne et al., 2003). Decreased glycine modifying enzyme Gnmt expression is consistent with a deregulated glycine metabolism and lower Acyl-CoA synthetase Acsl3 expression also implies an inefficient phase 2 metabolism. Finally, glutathione-S transferase Gstk1 plays a major role in phase 2 detoxification participating in the maintenance of oxidative balance (Deponte, 2013). The balance between phase 1 and phase 2 enzymes is critical for proper detoxification. Food, smoking, alcohol consumption, age and genetics are known factors impacting detoxification enzymes producing intermediary harmful metabolites faster than they can be detoxified, ultimately leading to increased oxidative stress and tissue damage (Liska, 1998). Our data suggest that sucralose and/or acesulfame-K also belong to these pathogenic factors. This is consistent with a previous study showing an association of sucralose consumption and microbiome-dependent liver inflammation (Bian et al., 2017a). Since our findings were most impressive in the pups, we speculate that the pre- and postnatal period represent a highly vulnerable developmental phase (Abou-Donia et al., 2008). At 20-days postpartum, pups are still developing their detoxification systems and metabolism. At this critical developmental stage exposure to NNS might be more consequential. Also, 20-days-old pups have a limited nutritional influx, mostly breast milk. At weaning, the increased variety of nutrients may further challenge metabolic detoxification. Indeed, endotoxins, end products of metabolism, bacterial as well as intermediary metabolites might accumulate, creating a toxic environment impossible to overcome.

### Microbiome Alterations in NNS Pups

Following pre- and post-natal exposure to NNS, pups have a dramatically different microbial composition, which is not due to alteration of mother’s microbiome. Indeed, mothers and pups’ microbiome are expected to look alike, consequences of a high microbe exchange through vaginal and physical contact, breastfeeding and murine coprophagic behavior (Faith et al., 2013; Bäckhed et al., 2015).

Studies in adult mice have previously shown NNS’ detrimental effects on the intestinal microbiome, mostly increases in *clostridiales*, *lactobacilli*, and *enterobacteria* (Abou-Donia et al., 2008; Cowan et al., 2013; Palmnäs et al., 2014; Suez et al., 2014; Bian et al., 2017b). A bacteriostatic effect has also been suggested in microbes in the oropharynx (Prashant et al., 2012). The microbiome signature of the NNS pups is a large increase in firmicutes combined with a marked depletion of Verrucomicrobia, represented exclusively by *A. muciniphila*. Both of these alterations are clinically relevant and have been associated with increased incidence of obesity and/or metabolic syndrome in rodents and humans (Ley et al., 2005; Hansen et al., 2012; Schneeberger et al., 2015). The increase in firmicutes observed in NNS pups is mainly due to the *clostridiales* order, the *lachnospiraceae* family and *oscillospira* genus. Those three *clostridia* class members are known to participate in normal metabolism of bile acids, carbohydrates and amino acids and participate in colonic epithelium health by producing short chain fatty acids (Donohoe et al., 2011). Therefore, increases in *clostridia* can influence colonocyte homeostasis. Interestingly, increases in exposed pups’ phospholipids and sphingolipids also are consistent with an increase gut cell proliferation.

*Akkermansia muciniphila*, depleted in NNS pups, is a beneficial bacterium associated with a young and lean metabolism, inversely correlated with fat mass gain, type 1 diabetes and Inflammatory bowel syndrome (Ley et al., 2005; Hansen et al., 2012; Schneeberger et al., 2015). A decrease in *A. muciniphila* was previously observed in response to long saccharin exposure in adult rats (Suez et al., 2014). Interestingly, *A. muciniphila* encodes a GlcNAc-6-sulfatase and the reduction in *A. muciniphila* in the NNS-pups’ gut is consistent with the abundance of GlcNAc-6-Sulfate in NNS pups’ feces. *In vitro* culture of *A. muciniphila* is not affected by the presence of NNS but the colonization is clearly deficient in the pups. Therefore, the improper colonization of *A. muciniphila* may be the result of a change in the mucin profile, fucosylated milk oligosaccharides and/or gut pH due to changes in metabolites, microbe abundance or inflammation status (Aakko et al., 2017;Van Herreweghen et al., 2017).

Metabolomics analysis is also suggestive of substantial gut microbiome alterations. Among the deregulated metabolites, N-formyl-methionine is used for protein synthesis in bacteria only (Gottschalk, 2012). Secondary bile acids are also exclusively produced by gut microbes from primary bile acid release in the GI tract. A decrease in secondary bile acids similar to ours, has been associated with decreased fasting glucose in mice (Kuno et al., 2018). Heme breakdown products biliverdin and bilirubin are also transformed by microbes to produce urobilinogen. From dietary aromatic compounds, the gut microbiome also metabolizes benzoate, the primary source of hippurate. Finally, gut microbes convert natural isoflavone glycosides into daidzein and genistein, which are readily absorbed. Gut microbiome function is also tightly linked to detoxification mechanisms. Indeed, microbial metabolites are detoxified by the host (Spanogiannopoulos et al., 2016) and in a complementary fashion, the gut microbiota transforms drugs and bile acids (Wahlström et al., 2016). Therefore, improper detoxification might be the result or the consequence of microbiome dysbiosis.

## Conclusion

In conclusion, pre- and post-natal exposure to sucralose and acesulfame-K via maternal ingestion causes marked metabolic and microbiome alterations in the pups (Figure 5E), which could give rise to future metabolic disease. This study adds to the growing list of negative effects linked to NNS consumption. Given that the perinatal period is a critical developmental stage for the nascent microbiome and emerging detoxification systems in the rodent and human neonate alike, our study defines potentially adverse consequences of early NNS exposure.

## Ethics Statement

This study was carried out in accordance with the recommendations of NIH guideline for animal use, Animal Care and Use Committee (NIH). The protocol was approved by the NIDDK Animal Care and Use Committee (NIH) under the protocol number #K023-LCBB-16.

## Author Contributions

SO-VS designed and performed the experiments and wrote the manuscript. JH and KR supervised the experiments and revised the manuscript.

## Conflict of Interest Statement

The authors declare that the research was conducted in the absence of any commercial or financial relationships that could be construed as a potential conflict of interest.

## Footnotes

1 https://github.com/stephvanstich

2 https://osf.io/z2mwr/?view_only=c4c951d59c91421cbec5ed9a07bd0f21

## Abbreviations

ADI: Acceptable daily intake as specified by the FDA
ADI1x: daily dose at upper limit of ADI
ADI2x: daily dose at twice the upper limit of the ADI
Ctrl, ADI1x, ADI2x or NNS mothers: Mothers fed either a Ctrl, ADI1x ADI2x or one of the ADI diet during pregnancy and lactation
Ctrl, ADI1x, ADI2x or NNS pups: Pups whose mother where fed either a Ctrl, ADI1x ADI2x or one of the ADI diet during pregnancy and lactation
FDA: Food and Drug Administration
NNS: Non-Nutritive Sweeteners
