# Supplementary figures and images for: Maternal Exposure to Non-nutritive Sweeteners Impacts Progeny’s Metabolism and Microbiome
Source: Front Microbiol. 2019 Jun 20;10:1360. doi: 10.3389/fmicb.2019.01360 (PMC6595049; doi:10.3389/fmicb.2019.01360)

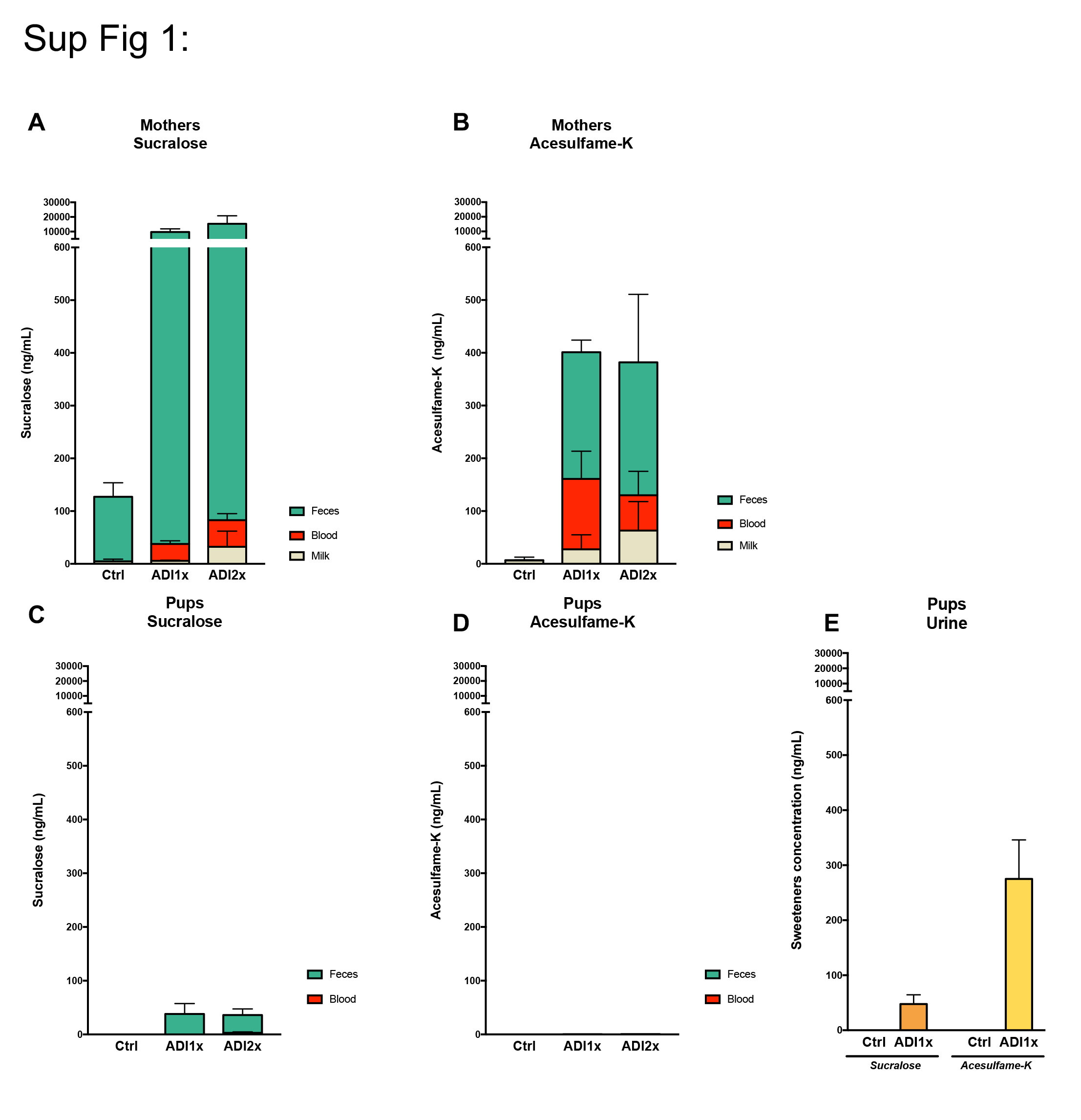

Supplement: Supplementary file 9 [file Image_1.JPEG]

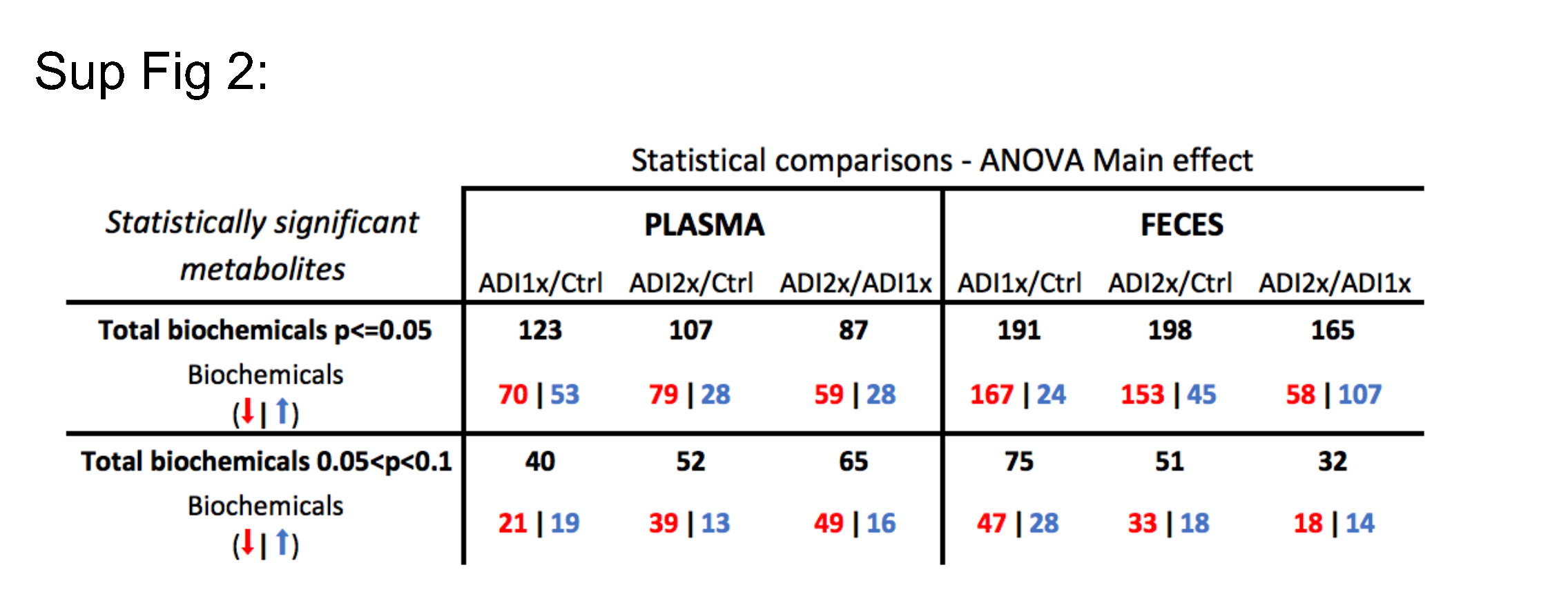

Supplement: Supplementary file 10 [file Image_2.JPEG]

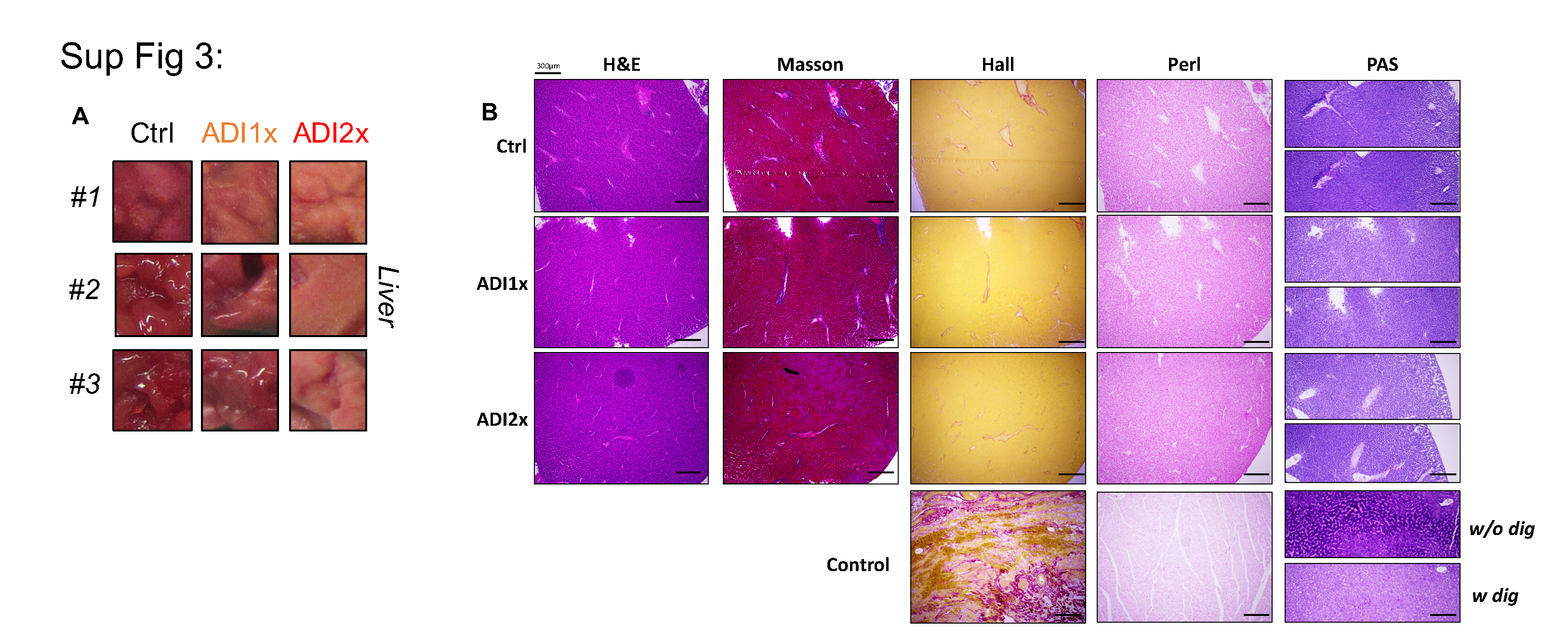

Supplement: Supplementary file 11 [file Image_3.jpg]

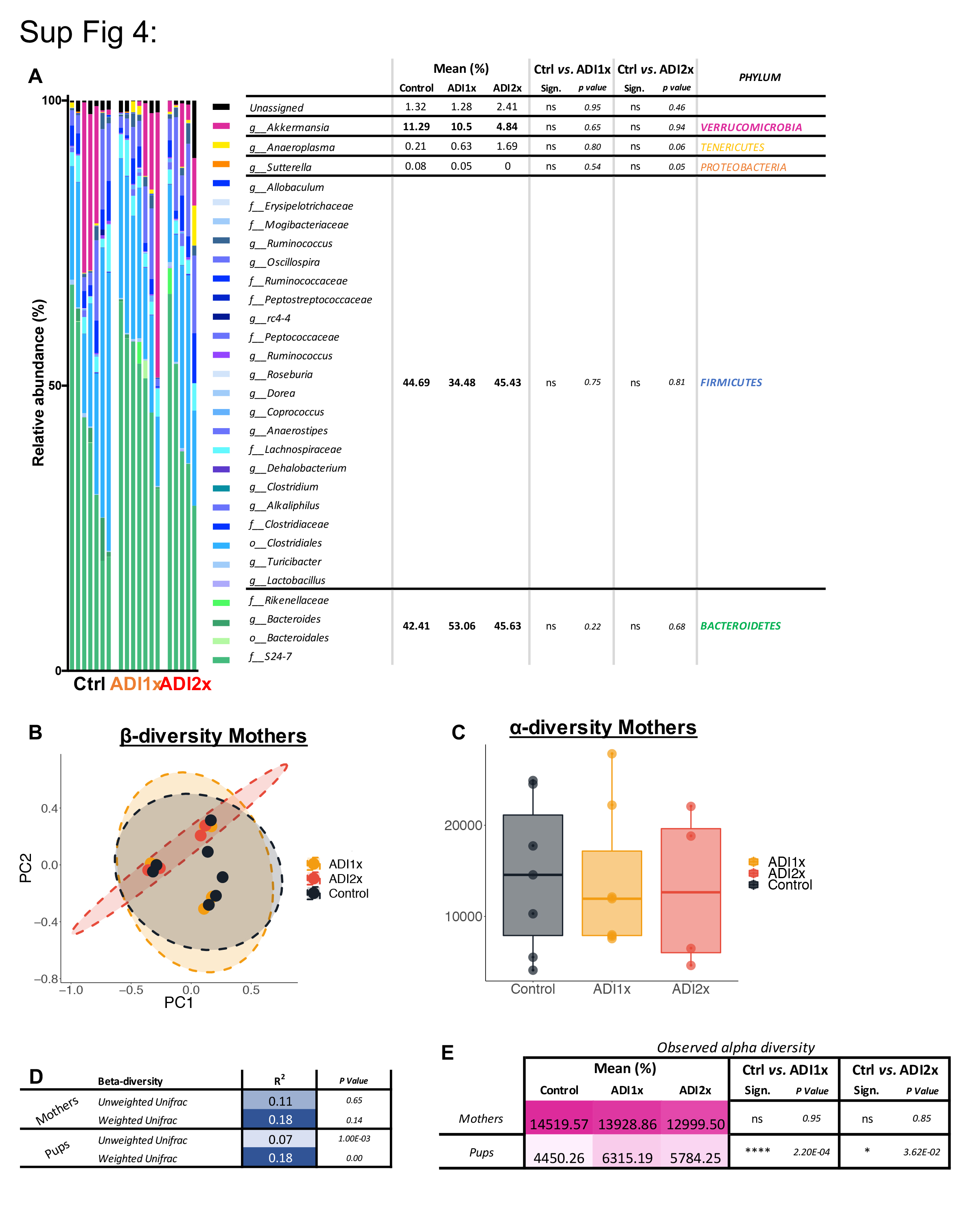

Supplement: Supplementary file 12 [file Image_4.JPEG]

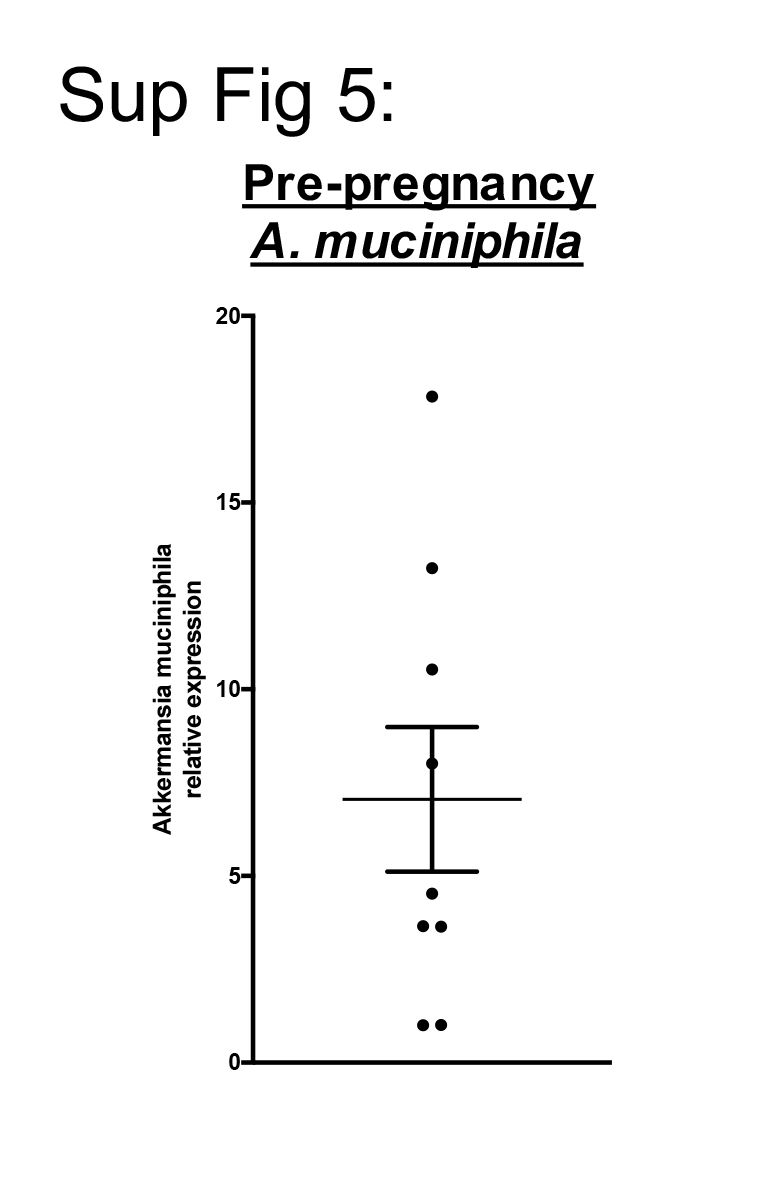

Supplement: Supplementary file 13 [file Image_5.JPEG]
