# Supplementary material for: Maternal Exposure to Non-nutritive Sweeteners Impacts Progeny’s Metabolism and Microbiome
Source: Front Microbiol. 2019 Jun 20;10:1360. doi: 10.3389/fmicb.2019.01360 (PMC6595049; doi:10.3389/fmicb.2019.01360)
Supplement: Supplementary file 14 [file Data_Sheet_1.PDF]

Supplementary Figures

Fig. S1. Sucralose and acesulfame-K are transmitted to the pups through breast milk. (A) Control, ADI1x and ADI2x mothers' sucralose concentrations in feces, blood and breast milk analyzed by LC-MS. **(B)** Control, ADI1x and ADI2x mothers' acesulfame-K concentrations in feces, blood and breast milk analyzed by LC-MS. **(C)** Control, ADI1x and ADI2x pups' sucralose concentrations in feces and blood analyzed by LC-MS. **(D)** Control, ADI1x and ADI2x pups' acesulfame-K concentrations in feces and blood analyzed by LC-MS. **(E)** Control and ADI1x pups' sucralose and acesulfame-K concentrations in urine analyzed by LC-MS.

Fig. S2. Summary table of deregulated metabolites in pups' plasma and feces at d20.

Fig. S3. NNS pups liver picture and histochemistry. (A) Images of 20-day old pups' livers obtained at sacrifice (n=3). **(B)** Histologic staining of 20-day old pups' livers (n=6). Only 3 livers are shown on this picture but staining are representative of at least 6 pups/ condition. PA (Periodic acid–Schiff) was performed with or without prior digestion of glycogen. Control slide for each staining are: Hall staining on liver with gall bladder; Perl staining on heart tissue and PAS staining on adult liver.

Fig. S4. Microbiome analysis demonstrates minor change in NNS fed mothers. (A) 16S sequencing of control, ADI1x and ADI2x gut microbiota of mothers. Mean, significance and p-values are represented at the phylum level on the right panel (Kruskal-Wallis test) (n=4). *g_*: genus; *f_*: family; *o_*: order. **(B)** Beta-diversity analysis (principal coordinate analysis (PCoA-unweighted unifrac)) of control, ADI1x and ADI2x microbiome sequencing (n=4). **(C)** Alpha-diversity analysis (observed operational taxonomic units (OTUs)) of control, ADI1x and ADI2x microbiome sequencing (n=4). **(D)** ADONIS test result of the weighted and unweighted Unifrac beta-diversity. **(E)** The changes observed in mothers and pups in alpha-diversity (Kruskal-Wallis test) (n=24). Significance values: **** $p<0.001$; *** $p<0.005$; ** $p<0.01$; * $p<0.05$.

Fig. S5. Pre-pregnancy *Akkermansia muciniphila* levels highlight variable colonization rate. A. muciniphila/16S level analyzed by qPCR in mothers' feces at d-3 of the experiment (n=9).

Table S1. Dosage of sucralose and Acesulfame-K in mothers and pups shows transmission of NNS.

Table S2. Mother and pup numbers with litter size and gender.

Table S3. Plasma metabolomics heat map of control, ADI1x and ADI2x pups.

Table S4. Feces metabolomics heat map of control, ADI1x and ADI2x pups.

Table S5. Phase 1 metabolism transcriptomics (qPCR array) Heat map.

Table S6. Phase 2 metabolism transcriptomics (qPCR array) Heat map.

Table S7. Microbiome 16S sequencing relative abundance in control, ADI1x, ADI2x pups.

Table S8. Microbiome 16S sequencing relative abundance in control, ADI1x, ADI2x mothers.
